# Performance of Five Thai Versions of Sarcopenia Screening Questionnaires (SARC-F, SARC-CalF, MSRA-7, MSRA-5, and Modified MSRA-5) in Thai Rheumatoid Arthritis Patients: A Cross-Sectional Study

**DOI:** 10.3390/jcm14228029

**Published:** 2025-11-12

**Authors:** Wanitcha Gumtorntip, Phichayut Phinyo, Nuntana Kasitanon, Worawit Louthrenoo

**Affiliations:** 1 Division of Rheumatology, Department of Internal Medicine, Faculty of Medicine, Chiang Mai University, Chiang Mai 50200, Thailand; wanitcha.ch@cmu.ac.th (W.G.); nuntana.k@cmu.ac.th (N.K.); 2 Center of Clinical Epidemiology and Clinical Statistics, Faculty of Medicine, Chiang Mai University, Chiang Mai 50200, Thailand; phichayut.phinyo@cmu.ac.th; 3 Department of Biomedical Informatics and Clinical Epidemiology, Faculty of Medicine, Chiang Mai University, Chiang Mai 50200, Thailand

**Keywords:** rheumatoid arthritis, sarcopenia, questionnaires, validation

## Abstract

**Background/Objectives:** The external validity of sarcopenia screening questionnaires in the elderly has been examined in several conditions but rarely evaluated in patients with rheumatoid arthritis (RA). This study aimed to determine the performance of five Thai versions of sarcopenia screening questionnaires (SARC-F [Strength, Assistance with walking, Rising from a chair, Climbing stairs, and Falls], SARC-CalF [SARC-F plus calf circumference], MSRA [Mini Sarcopenia Risk Assessment]-7, MSRA-5, and modified MSRA-5 questionnaires) in Thai RA patients, and evaluate the correlations among these instruments. **Methods**: In this cross-sectional study, consecutive adult RA patients (aged ≥20 years) from an outpatient rheumatology clinic completed the five sarcopenia screening questionnaires listed above. Sarcopenia was defined according to criteria of the 2019 Asian Working Group for Sarcopenia (AWGS). Appendicular skeletal muscle mass, grip strength, and physical performance were assessed using bioelectrical impedance analysis, a hand dynamometer, and a 6 m gait speed test, respectively. The cut-off values used for each sarcopenia screening questionnaire were pre-specified according to their respective established thresholds. **Results**: Of 299 RA patients (89.0% female, mean age of 61.3 ± 11.6 years, median [interquartile range] disease duration of 12.8 [8.2, 20.0] years), 37.5% and 27.4% of them had sarcopenia and severe sarcopenia, respectively. The areas under the receiver operating characteristic (ROC) curve for the SARC-F, SARC-CalF, MSRA-7, MSRA-5, and modified MSRA-5 questionnaires were 0.60, 0.74, 0.65, 0.62, and 0.65, respectively, with sensitivities of 34.8%, 73.2%, 77.7%, 68.8%, and 72.3% and specificities of 84.5%, 75.4%, 51.3%, 55.1%, and 58.3%, respectively. SARC-F demonstrated moderate correlations with the other questionnaires: SARC-CalF (r = 0.57), MSRA-7 (r = −0.52), MSRA-5 (r = −0.55), and modified MSRA-5 (r = −0.65), all with a *p*-value of <0.001. **Conclusions**: Sarcopenia is common among Thai RA patients. SARC-CalF had the best balance of sensitivity and specificity and is likely the most suitable sarcopenia screening questionnaire for Thai RA patients.

## 1. Introduction

According to the 2019 Asian Working Group for Sarcopenia (AWGS), sarcopenia is defined as low muscle mass plus either low muscle strength or low physical performance, and severe sarcopenia is diagnosed when all three components are present simultaneously [1]. The pathogenesis of sarcopenia is not fully understood, but several factors are involved, including malnutrition, hormone changes, increased inflammatory cytokine levels (TNFα, IL-1α and IL-6), and oxidative stress. These factors lead to increased muscle degradation, atrophy, and loss of muscle mass [2]. In addition, sarcopenia is linked to reductions in mobility, resting energy expenditure, and increased fat mass [3]. It also leads to decreased muscle function, increased risk of frailty, muscle injury, falls, and loss of independence [4]. Sarcopenia can be classified as primary sarcopenia, which is associated with aging, and secondary sarcopenia, which is associated with chronic inflammatory or chronic conditions [5]. 

Rheumatoid arthritis (RA) is a chronic inflammatory condition that primarily affects the joints. Sarcopenia in RA is common. In a meta-analysis, the prevalence of sarcopenia in RA patients was reported to range from 10.3% to 50.0%, with a pooled estimate of 25.4% [6]. This variation in prevalence may be influenced by differences in geographic region, age, disease duration, and the diagnostic criteria used to define sarcopenia. A study conducted in Central Thailand using the 2019 AWGS criteria found a sarcopenia prevalence of 26.4% [7]. RA patients with sarcopenia are at increased risk of falls, fractures, and worsened cardiometabolic status. They also have poor quality of life [8]. As sarcopenia has a negative impact on health in RA patients, identifying and diagnosing this condition early is crucial in enabling appropriate intervention and prevention.

Several questionnaires have been developed and used to screen for sarcopenia in the elderly [9]. In 2013, Malmstrom and Morley developed a questionnaire called SARC-F (Strength, Assistance with walking, Rising from a chair, Climbing stairs, and Falls) and suggested its use as a first step for screening sarcopenia [10]. However, although it has high specificity (82.8–98.8%), its sensitivity is relatively low (4.8–35.6%) [11,12]. In 2016, the SARC-F was modified to the SARC-CalF by adding a calf circumference (CC) item, which improved sensitivity (66.7%) and yielded acceptable specificity (82.9%) [13]. Later, in 2017, two Mini Sarcopenia Risk Assessment questionnaires (MSRA-7 and MSRA-5) were developed. The MSRA questionnaire is composed of general and dietary assessments [14]. These questionnaires have been tested in the elderly in community, hospital, and nursing home settings [10,11,14,15,16]. The MSRA-7 and MSRA-5 have been studied in the Thai population, showing 72.3% and 61.5% sensitivity, respectively, and 43.0% and 67.4% specificity, respectively [17]. Due to its relatively low sensitivity, the original Thai MSRA-5 questionnaire was revised to develop a modified version (modified MSRA-5) [17]. While this new version retains the original five items, it adjusts the scoring for questions regarding hospitalizations and daily meal frequency. These modifications have been shown to improve the sensitivity of the questionnaire for detecting sarcopenia in the elderly compared to the original MSRA-5.

As sarcopenia can occur in multiple chronic conditions, these questionnaires have been validated across diverse patient populations. Of these, SARC-F is possibly the most widely used and has been validated in patients with cancer [18,19], diabetes mellitus [20], stroke, chronic liver disease [21,22,23], dialysis [24,25], stroke [26], chronic heart failure [27], and systemic sclerosis [28]. In contrast, the MSRA-7 and MSRA-5 have been validated in a limited number of studies, primarily in cancer settings [29,30,31]. Unfortunately, their use in RA patients is very limited [32,33].

This study aimed to determine the performance of Thai versions of the SARC-F, SARC-CalF, MSRA-7, MSRA-5 and modified MSRA-5 sarcopenia screening questionnaires in Thai RA patients. It also aimed to determine the correlation among them. We hypothesized that these Thai versions of sarcopenia screening questionnaires would have acceptable diagnostic performance, with significant correlations in detecting sarcopenia among Thai RA patients. Although sarcopenia is commonly observed in the elderly, RA patients may develop it at a younger age. This could be due to disease severity, presence of inflammatory mediators, and limited mobility, all of which contribute to muscle loss. Therefore, all RA patients were included in this study regardless of their age.

## 2. Materials and Methods

### 2.1. Patients

This cross-sectional study recruited consecutive adult RA patients (age ≥20 years), who attended the out-patient rheumatology clinic of Chiang Mai University Hospital (a 1200-bed hospital in northern Thailand) between May and November 2023. All of them fulfilled the 2010 American College of Rheumatology (ACR)/European League Against Rheumatism (EULAR) classification criteria for RA [34]. In addition, they had to be ambulatory and able to both walk independently and perform a handgrip. Exclusion criteria included concomitant inflammatory joint diseases (e.g., spondyloarthropathies, systemic sclerosis, idiopathic inflammatory myopathy, systemic lupus erythematosus, vasculitis), severe hand deformities precluding handgrip assessment, kyphotic deformity precluding bioelectrical impedance analysis (BIA), pregnancy, and the presence of a cardiac pacemaker or other implanted devices.

### 2.2. Outcomes Measurement

After the patients had signed the consent form, their demographic data, underlying conditions, comorbidities, and current medications were reviewed. Disease activity was determined using the Disease Activity Score (DAS), with 28 joint counts, and erythrocyte sedimentation rate (ESR) (DAS28-ESR) [35] by a rheumatologist (WG). All of the patients completed the five Thai versions of sarcopenia screening questionnaires: SARC-F, SARC-CalF, MSRA-7, MSRA-5, and the modified MSRA-5. The SARC-F, MSRA-7, MSRA-5, and modified MSRA-5 questionnaires, which were translated into the Thai language and validated in elderly Thai patients [17].

The SARC-F questionnaire consisted of five items, and the total score ranged from 0 to 10 points [10]. The evaluation comprised five questions that evaluated the clinical symptoms commonly associated with sarcopenia, such as the ability to lift and carry 10 pounds of weight, walk across a room, transfer from a chair to bed, and climb a flight of 10 steps, as well as experience any falls in the past year. The SARC-CalF was created by adding the CC to the SARC-F questionnaire [13]. The score of SARC-CalF is similar to that of SARC-F, but with an additional score for CC. Based on the 2019 AWGS criteria, an additional 10 points were assigned to male and female individuals with CC measurements of <34 cm and <33 cm, respectively, according to the cut-off values for possible sarcopenia [1].

The MSRA-7, MSRA-5 and modified MSRA-5 consisted of seven, five and five questions, respectively, which covered age, amount of physical activity, regular meal consumption, dairy product and protein intake, number of hospitalizations, and weight loss during the past year [14,17]. The cumulative scores for the MSRA-7, MSRA-5 and modified MSRA-5 ranged between 0–40, 0–60 and 0–34 points, respectively. The cut-off points suggestive of sarcopenia for the SARC-F, SARC-Calf, MSRA-7, MSRA-5 and modified MSRA-5 questionnaires were ≥4, ≥11, ≤30, ≤45 and ≤30, respectively [10,14,17].

The BIA (Tanita-RD545), hand dynamometer, and gait speed test were used to determine appendicular skeletal muscle mass, handgrip strength, and physical performance, respectively. For the BIA assessment, the subject stands barefoot on the device in an upright position and grips the handles firmly until the measurement is complete. For the handgrip strength test, the patients sat with their elbow flexed at a 90-degree angle. The handgrip strength was measured three times on the dominant side using the Jamar Hydraulic Hand Dynamometer, with the mean value of three measurements used for analysis. Then, the patients were asked to walk 6 m three times at a regular speed from a moving start, without deceleration, to determine their gait speed. The average gait speed from the three measurements was used for analysis, and a manual stopwatch was employed. The CC was determined while the patients were standing by measuring the maximal circumference of both calves using a non-elastic tape without applying tension. The average of the two measurements was used for analysis. Patients completed the questionnaires themselves prior to performing the BIA, and grip strength and gait speed tests, which were assessed sequentially by an assessor (NW), who was blinded to the questionnaire scores to avoid bias.

In this study, sarcopenia was defined according to the 2019 AWGS criteria as a loss of muscle mass plus either reduced muscle strength or poor physical performance [1]. Low appendicular skeletal muscle mass was defined by a BIA of <7.0 kg/m^2^ and <5.7 kg/m^2^ in men and women, respectively. Low muscle strength was defined by a handgrip strength of <28.0 kg and <18.0 kg for men and women, respectively. Low physical performance was defined by a 6 m walk at <1.0 m/s. In addition, severe sarcopenia was defined as low muscle mass plus low muscle strength and low physical performance [1].

### 2.3. Statistical Analysis

The sample size was calculated based on the sensitivity of the SARC-F (21%) and specificity (93.7%) from a sarcopenia study in the Thai population [17]. Assuming that the SARC-F performs equally well in the RA population, with the same sensitivity and specificity, a sample size of 64 would be required to estimate the proportion of sensitivity, with 10% absolute precision and 95% confidence interval (95% CI). Assuming a sarcopenia prevalence in RA of 30%, a minimum of 214 RA patients would be required to achieve the target of 64 sarcopenia cases.

The STATA version 17 (StataCorp, College Station, TX, USA) was used for data analysis. Categorical data were presented as number (%), and continuous data as mean (standard deviation, SD) for normally distributed data or median and interquartile range (IQR) for non-normally distributed data. The Chi-square test was used to determine the difference among categorical variables, and Student’s *t*-test for comparing means between independent groups. Sensitivity, specificity, positive likelihood ratio (LR+), negative likelihood ratio LR–, and area under the receiver operating characteristic (ROC) curve were calculated. The diagnostic criteria for sarcopenia, according to the 2019 AWGS, were used as the reference standard. To compare the discriminatory performance of the screening tools, pairwise differences in ROC areas were evaluated using DeLong’s method, and *p*-values from these comparisons were adjusted using the Holm–Bonferroni method to control the family-wise error rate. Pearson’s correlation coefficient was used to assess the correlation between screening questionnaires. A *p*-value of <0.05 was considered statistically significant.

## 3. Results

### 3.1. Characteristics of the Patients Studied

Three hundred and twelve consecutive adult RA patients were invited to participate in this study; however, 13 were excluded due to being unable to walk (7 patients) or having significant hand abnormalities, which made them unable to grip tightly (6 patients). A total of 299 participants were included in the study. Their average age was 61.3 ± 11.6 years and 266 (89.0%) of them were female. The median (IQR) duration of RA was 12.8 (8.2, 20.0) years. Thirty-one patients (13.7%) had deformities in their hands or feet. Rheumatoid factor (RF) and anti-cyclic citrullinated peptide (anti-CCP) were positive in 77.6% and 85.2% of cases, respectively. The mean of Disease Activity Score, using 28 joint counts with ESR (DAS28-ESR), was 3.6 ± 0.9, and the mean body mass index (BMI) was 22.4 ± 3.8 kg/m^2^. Hypertension, dyslipidemia, osteoarthritis, osteoporosis, and diabetes mellitus were common comorbidities. The mean Charlson co-morbidities index was 3.0 ± 1.3. The RA patients were treated with methotrexate (88.3%), leflunomide (25.4%), sulfasalazine (7.7%), and glucocorticoids (62.5%) with a mean dosage of 4.2 mg/day, as well as non-steroidal anti-inflammatory drugs (13.4%). Biologic disease-modifying antirheumatic drugs were used in 4.4% of the RA patients. The characteristics of RA patients are shown in Table 1.

One hundred and twelve patients (37.5%, 95% CI: 32.4, 43.1) had sarcopenia, with 82 (27.4%, 95% CI: 22.1, 32.1) classified as having severe sarcopenia. Among the RA patients with sarcopenia, the mean appendicular skeletal muscle mass index, mean grip strength, and mean gait speed were 5.3 ± 0.6 kg/m^2^, 11.8 ± 4.9 kg, and 0.8 ± 0.3 m/s, respectively. The components of sarcopenia in the RA patients are shown in Table 2. There were no significant differences in medication use between sarcopenia and non-sarcopenia RA patients.

### 3.2. Performance of Five Thai Versions of SARC-F, SARC-CalF, MSRA-7, MSRA-5 and Modified MSRA-5 Questionnaires for Screening Sarcopenia

**Figure 1:**
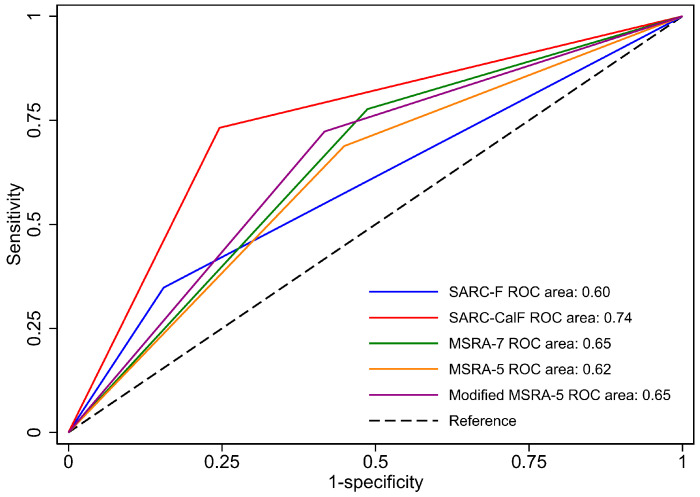
The receiver operating characteristic curves of Thai versions of SARC-F, SARC-CalF, MSRA-7, MSRA-5, and modified MSRA-5 questionnaires. Abbreviations: MSRA = Mini Sarcopenia Risk Assessment questionnaire; ROC = receiver operating characteristic; SARC-CalF = Strength, Assistance with walking, Rising from a chair, Climbing stairs, Calf circumference, and Falls questionnaire; SARC-F = Strength, Assistance with walking, Rising from a chair, Climbing stairs, and Falls questionnaire.

The efficacy of the questionnaire in identifying RA patients with sarcopenia is shown in Table 3. The sensitivity and specificity of each questionnaire were as follows: SARC-F (34.8% and 84.5%, respectively), SARC-CalF (73.2% and 75.4%, respectively), MSRA-7 (77.7% and 51.3%, respectively), MSRA-5 (68.8% and 55.1%, respectively), and modified MSRA-5 (72.3% and 58.3%, respectively). In addition, the questionnaires provided LR+, LR–, and ROC area values, as follows: SARC-F (2.3, 0.8 and 0.60, respectively), SARC-CalF (3.0, 0.4 and 0.74, respectively), MSRA-7 (1.6, 0.4 and 0.65, respectively), MSRA-5 (1.5, 0.6 and 0.62, respectively), and modified MSRA-5 (1.7, 0.5 and 0.65, respectively). The ROC area value for SARC-CalF was the highest among the questionnaires (Figure 1). The pairwise comparison of differences in ROC area (ΔROC area), using SARC-F as the reference screening tool, showed that the ΔROC area between SARC-F and SARC-CalF was −0.14 (95% CI: −0.20, −0.09), indicating that SARC-CalF had significantly higher discriminative ability. For the other tools, the differences were smaller: −0.05 (95% CI: −0.11, 0.01) for MSRA-7, −0.02 (95% CI: −0.08, 0.04) for MSRA-5, and −0.05 (95% CI: −0.11, 0.01) for modified MSRA-5.

### 3.3. Correlations Among the Screening Questionnaires

Correlations among the screening questionnaires for identifying sarcopenia in RA patients are shown in Table 4. The SARC-F showed a moderately positive correlation with SARC-CalF (r = 0.57) and moderately negative correlation with the MSRA-7 (r = −0.52), MSRA-5 (r = −0.55) and modified MSRA-5 (r = −0.65). The SARC-CalF demonstrated a low negative correlation with MSRA-7 (r = −0.39), and a moderately negative correlation with both the MSRA-5 (r = −0.44) and modified MSRA-5 (r = −0.49). The MSRA-7 showed a strong positive correlation with the MSRA-5 (r = 0.85) and modified MSRA-5 (r = 0.77). The strongest correlation was observed between MSRA-5 and modified MSRA-5 (r = 0.88). All of these correlations were highly significant, with a *p*-value of <0.001.

## 4. Discussion

This study found the prevalence of sarcopenia and severe sarcopenia to be 37.5% and 27.4%, respectively, thus showing these conditions as common among Thai RA patients. The ROC area of these five questionnaires (SARC-F, SARC-CalF, MSRA-7, MSRA-5, and modified MSRA-5) ranged between 0.60 and 0.74, with sensitivities and specificities ranging from 34.8% to 77.7% and 51.3% to 84.5%, respectively. This indicates that these instruments are acceptable but with variable accuracy as screening tools for sarcopenia in Thai RA patients. Among them, the SARC-CalF questionnaire demonstrated the most favorable sensitivity and specificity, as well as the highest LR+ and lowest LR–, suggesting that it is likely the best screening instrument among those evaluated. Additionally, SARC-F showed a moderate correlation with SARC-CalF, MSRA-7, MSRA-5, and the modified MSRA-5.

Although sarcopenia in RA patients has been widely studied and was recently reviewed in a systematic review and meta-analysis [36], the use of questionnaires for screening sarcopenia has rarely been reported. A study in Spain by Valencia-Muntalà et al. used the 2018 European Working Group on Sarcopenia in Older People (EWGSOP)-2 criteria for diagnosing sarcopenia and found a prevalence of 16.4% [32], and the SARC-F questionnaire in that study showed sensitivity and specificity of 100% and 75%, respectively. The authors concluded that the SARC-F questionnaire was effective in predicting sarcopenia in RA patients. In contrast, a study in Russia by Dobrovolskaya et al. also used the 2018 EWGSOP-2 criteria, and reported a sarcopenia prevalence of 24%. In that study, the patients met the SARC-F criteria, with a sensitivity and specificity of 30% and 41%, respectively [33]. The 37.5% prevalence of sarcopenia observed in this study, which was higher than that reported in the Spanish and Russian studies, was not unexpected. This higher prevalence may be explained by the fact that this study used the 2019 AWGS criteria, specifically developed for Asian populations with generally smaller body sizes, whereas the Spanish and Russian studies, which comprised primarily Caucasian participants, utilized the 2018 EWGSOP-2 criteria [5]. The 2019 AWGS criteria defines sarcopenia as low appendicular skeletal muscle mass and either low muscle strength or low physical performance (cut-off values: muscle mass <7.0 kg/m^2^ for men and <5.7 kg/m^2^ for women; handgrip strength <28.0 kg for men and <18.0 kg for women; and gait speed of <1.0 m/s), and severe sarcopenia is diagnosed when these three components occur simultaneously [1]. In contrast, the 2018 EWGSOP-2 criteria focuses on low muscle strength as a key characteristic of sarcopenia (low muscle quantity and quality) for the diagnosis of sarcopenia and poor physical performance (gait speed test) to indicate severe sarcopenia (cut-off values: muscle mass <7.0 kg/m^2^ for men and <5.5 kg/m^2^ for women; handgrip strength <27 kg for men and <16 kg for women; and gait speed ≤0.8 m/s) [5]. In addition, the 2018 EWGSOP-2 criteria emphasizes a stepwise diagnostic approach to determine the severity of sarcopenia, while the 2019 AWGS criteria use all these measures to diagnose at the same time. Regarding sarcopenia screening tools, the SACF-F demonstrated slightly higher sensitivity but twice the specificity than the Russian study [33] and markedly lower sensitivity but slightly higher specificity compared to the Spanish study [32]. Interestingly, the Spanish study reported 100% sensitivity with the SARC-F questionnaire. Therefore, in addition to the differences in the diagnostic criteria used to determine the prevalence of sarcopenia, factors beyond ethnicity and baseline characteristics may also influence the performance of these screening tools.

In this study, the performance of the Thai screening questionnaires (the SARC-F, MSRA-7, MSRA-5, and modified MSRA-5) in RA patients showed sensitivity, specificity, and ROC area comparable to those of the original translations, which were validated for sarcopenia in elderly Thai individuals by Akarapornkrailert et al. (Table 5) [17]. These findings indicate that these Thai versions of sarcopenia screening questionnaires can also be used in Thai RA patients. Unfortunately, the SARC-CalF was not included in the original translation and validation study [17].

This study found that the SARC-CalF questionnaire provided the highest area under the ROC when compared with the other questionnaires, thus indicating its suitability as a screening tool for predicting sarcopenia in Thai RA patients. Kawakami et al. demonstrated a positive correlation between CC and both appendicular skeletal muscle mass and skeletal muscle index in the elderly Japanese population [37]. However, the optimal cut-off points for CC among different ethnic groups were not clearly established. Although this study used the recommended CC cut-off point from the 2019 AWGS [1], it might not directly apply to the Thai population. It should be noted that the lower extremity joints are also commonly affected in RA patients, and chronic inflammation combined with limited joint mobility could lead to calf muscle atrophy, resulting in decreased CC. Prolonged steroid use in RA patients can also contribute to reduced CC by promoting muscle breakdown and wasting, ultimately resulting in muscle atrophy [38]. This could explain why the SARC-CalF performed better than the SARC-F in sarcopenia screening among RA patients. Unfortunately, studies that evaluated other sarcopenia questionnaires for screening sarcopenia in RA patients remain extremely limited, and more studies are needed to confirm these findings.

This study found only a moderate correlation between the SARC-F (and SARC-CalF) and MSRA (MSRA-7, MSRA-5, and modified MSRA-5) questionnaires. This might be attributed to the fact that the SARC-F and SARC-CalF primarily focus on domains related to physical function (muscle strength, physical performance, falls, and muscle mass), whereas the MSRA questionnaires assess various domains including age, hospitalization, comorbidities, and dietary factors. Therefore, the limited correlation between these instruments might reflect differences in their underlying constructs, in that the SARC-F/SARC-CalF questionnaires predominantly capture functional impairment (convergent validity within strength/performance constructs), while MSRA questionnaires emphasize risk factors and health history (discriminant validity from non-overlapping constructs).

There were some limitations in this study. First, there is no universally accepted gold standard method for CC measurement. The participant’s posture during CC measurement, whether standing or sitting, may potentially influence the results. However, several studies recommend measuring CC in a standing position, as it provides greater accuracy and reduces the risk of overestimation [39,40]. In this study, CC was measured in the standing position to help ensure more accurate results. Second, this study used BIA to assess muscle mass rather than more accurate methods such as dual X-ray absorptiometry (DXA), magnetic resonance imaging (MRI) or computed tomography (CT). Factors such as hydration status and recent food intake can affect BIA measurements and potentially introduce variability. However, in this study, all measurements were performed in the mid-morning without fasting. Therefore, these factors should not have contributed to the high variability observed in BIA measurements. A recent study in Thailand found that the BIA instrument (Tanita RD-545), as also used in this study, had a very high level of agreement with a DXA machine (GE Lunar iDXA) in the measurement of appendicular skeletal muscle mass, with an intraclass correlation coefficient of 0.908 [41]. Nevertheless, BIA has been proposed as an alternative to DXA and is recommended by recognized sarcopenia working groups as an effective method for assessing muscle mass in clinical settings [1,5]. 

Despite these limitations, this study also had strengths. The reference measurements including BIA assessment, hand grip strength, gait speed test, and CC measurement were performed in all of the participants by one study coordinator (NW), who was trained by a supervisor (WG), with consistent results. Using a single trained assessor minimized potential inter-rater variability, thereby enhancing the reliability of the study outcomes. Additionally, this study was probably the first to evaluate the correlations among multiple sarcopenia screening questionnaires in RA patients. The results from this study could serve as an initial benchmark for future studies by examining the use of sarcopenia screening questionnaires in RA patients within Asian populations.

Further research should explore the performance of these questionnaires in early RA or among patients with severe or treatment-refractory RA. The impact of, or correlation between, sarcopenia and RA outcomes, including disease activity, medication response, relapse rates, and overall severity, should also be investigated. Moreover, optimal therapeutic strategies for RA patients with sarcopenia, such as selecting medication, dosing, and the need for multidisciplinary management to improve patient care, should be explored.

## 5. Conclusions

Sarcopenia is commonly found in Thai RA patients. These five Thai sarcopenia screening questionnaires (SARC-F, SARC-CalF, MSRA-7, MSRA-5, and modified MSRA-5) are acceptable screening tools for sarcopenia in Thai RA patients, though their accuracy varies. Of these, the SARC-CalF questionnaire is likely the best screening instrument among those evaluated.

## Figures and Tables

**Table 1:** Characteristics of rheumatoid arthritis patients.

Characteristics	N = 299
Sex (Female)	266 (89.0%)
Age (years)	61.3 ± 11.6
BMI (kg/m^2^)	22.4 ± 3.8
Calf circumference (cm)	31.8 ± 3.6
Underlying disease	
Hypertension	109 (36.5%)
Dyslipidemia	115 (38.5%)
Diabetes mellitus	33 (11.0%)
Osteoporosis	60 (20.0%)
Charlson comorbidity index	3.0 ± 1.3
Smoking	29 (9.7%)
Disease duration (years)	12.8 (8.2, 20.0)
RF seropositivity	232 (77.6%)
Anti-CCP antibody seropositivity	254 (85.2%)
DAS28-ESR	3.6 ± 0.9
Current medication	
NSAIDs	40 (13.4%)
Glucocorticoid	187 (62.5%)
Methotrexate	264 (88.3%)
Hydroxychloroquine	19 (6.4%)
Sulfasalazine	23 (7.7%)
Leflunomide	76 (25.4%)
bDMARDs	13 (4.4%)

Data are presented as mean ± standard deviation, median (interquartile range), or n (%). Abbreviations: Anti-CCP = Anti-cyclic citrullinated peptides; bDMARDs = biological disease-modifying antirheumatic drugs; BMI = body mass index; DAS28 = Disease Activity Score-28; ESR = erythrocyte sedimentation rate; NSAIDs = non-steroidal anti-inflammatory drugs; RF = rheumatoid factor.

**Table 2:** Components of sarcopenia in rheumatoid arthritis patients.

	Sarcopenia N = 112 (37.46%)	Non-Sarcopenia N = 187 (62.54%)	*p*-Value
Sarcopenia AWGS			
Skeletal muscle mass index (kg/m^2^)	5.3 ± 0.6	6.7 ± 0.8	<0.001
Grip strength (kg)	11.8 ± 4.9	16.1 ± 6.8	<0.001
Gait speed (m/s)	0.8 ± 0.3	0.9 ± 0.3	<0.001
Sarcopenia questionnaire			
SARC-F	2 (0.5, 5)	1 (0, 2)	<0.001
SARC-CalF	12 (10, 15)	3 (0, 10)	<0.001
MSRA-7	27.5 ± 6.6	31.8 ± 5.9	<0.001
MSRA-5	40.9 ± 13.1	50.0 ± 10.7	<0.001
Modified MSRA-5	20.3 ± 10.3	27.5 ± 8.1	<0.001

Data are presented as mean ± standard deviation, median (interquartile range), or n (%). Abbreviations: AWGS = Asian Working Group for Sarcopenia; MSRA = Mini Sarcopenia Risk Assessment questionnaire; SARC-CalF = Strength, Assistance with walking, Rising from a chair, Climbing stairs, Calf circumference, and Falls questionnaire; SARC-F = Strength, Assistance with walking, Rising from a chair, Climbing stairs, and Falls questionnaire.

**Table 3:** Performance of Thai versions of SARC-F, SARC-CalF, MSRA-7, MSRA-5 and modified MSRA-5 questionnaires in rheumatoid arthritis patients.

Test and Cut-Off Value	Rheumatoid Arthritis Patients			Sensitivity (95% CI)	Specificity (95% CI)	LR+ (95% CI)	LR– (95% CI)	ROC Area (95% CI)	*p*-Values *
	Total N (%)	Sarcopenia N (%)	Non-Sarcopenia N (%)						
SARC-F									
≥4	68 (22.7)	39 (34.8)	29 (15.5)	34.8% (26.1, 44.4)	84.5% (78.5, 89.4)	2.3 (1.5, 3.4)	0.8 (0.7, 0.9)	0.60 (0.55, 0.65)	Reference
<4	231 (77.3)	73 (65.2)	158 (84.5)						
SARC-CalF									
≥11	128 (42.8)	82 (73.2)	46 (24.6)	73.2% (64.0, 81.1)	75.4% (68.6, 81.4)	3.0 (2.3, 3.9)	0.4 (0.3, 0.5)	0.74 (0.69, 0.79)	<0.001
<11	171 (57.2)	30 (26.8)	141 (75.4)						
MSRA-7									
≤30	178 (59.5)	87 (77.7)	91 (48.7)	77.7% (68.8, 85.0)	51.3% (43.9, 58.7)	1.6 (1.3, 1.9)	0.4 (0.3, 0.6)	0.65 (0.59, 0.70)	0.460
>30	121 (40.5)	25 (22.3)	96 (51.3)						
MSRA-5									
≤45	161 (53.9)	77 (68.8)	84 (44.9)	68.8% (59.3, 77.2)	55.1% (47.7, 62.3)	1.5 (1.3, 1.9)	0.6 (0.4, 0.8)	0.62 (0.56, 0.68)	1.000
>45	138 (46.2)	35 (31.3)	103 (55.1)						
Modified MSRA-5									
≤30	159 (53.2)	81 (72.3)	78 (41.7)	72.3% (63.1, 80.4)	58.3% (50.9, 65.4)	1.7 (1.4, 2.1)	0.5 (0.3, 0.7)	0.65 (0.60, 0.71)	0.223
>30	140 (46.8)	31 (27.7)	109 (58.3)						

Abbreviations: LR = Likelihood Ratio; MSRA = Mini Sarcopenia Risk Assessment questionnaire; ROC = receiver operating characteristic; SARC-CalF = Strength, Assistance with walking, Rising from a chair, Climbing stairs, Calf circumference, and Falls questionnaire; SARC-F = Strength, Assistance with walking, Rising from a chair, Climbing stairs, and Falls questionnaire. * Differences in ROC areas were tested using DeLong’s method, with Holm–Bonferroni adjustment for multiple comparisons, to control family-wise error.

**Table 4:** Correlation among screening questionnaires.

Correlation	SARC-F	SARC-CalF	MSRA-7	MSRA-5	Modified MSRA-5
SARC-F	1.00				
SARC-CalF	0.57 *	1.00			
MSRA-7	−0.52 *	−0.39 *	1.00		
MSRA-5	−0.55 *	−0.44 *	0.85 *	1.00	
Modified MSRA-5	−0.65 *	−0.49 *	0.77 *	0.88 *	1.00

* *p* < 0.001 Abbreviations: MSRA = Mini Sarcopenia Risk Assessment questionnaire; SARC-CalF = Strength, Assistance with walking, Rising from a chair, Climbing stairs, Calf circumference, and Falls questionnaire; SARC-F = Strength, Assistance with walking, Rising from a chair, Climbing stairs, and Falls questionnaire.

**Table 5:** Comparison of the performance of Thai sarcopenia screening questionnaires between elderly Thai patients and patients with rheumatoid arthritis.

	Akarapornkrailert et al. [17]	This Study
Population studied	Elderly Thai	Rheumatoid arthritis
Number of participants	286	299
Mean age in years	71.8 ± 7.8	61.3 ± 11.6
Muscle mass measurement instrument	BIA (MC-780), TANITA, Tokyo, Japan	BIA (RD545), TANITA, Tokyo, Japan
Prevalence of sarcopenia	22.7%	37.5%
Sarcopenia screening questionnaires		
SARC-F		
Sensitivity	21.5%	34.8%
Specificity	93.7%	84.5%
ROC area	0.58	0.60
SARC-CalF		
Sensitivity	NA	73.2%
Specificity	NA	75.4%
ROC area	NA	0.74
MSRA-7		
Sensitivity	72.3%	77.7%
Specificity	43.0%	51.3%
ROC area	0.58	0.65
MSRA-5		
Sensitivity	61.5%	68.8%
Specificity	67.4%	55.1%
ROC area	0.65	0.62
Modified MSRA-5		
Sensitivity	86.2%	72.3%
Specificity	43.4%	58.3%
ROC area	0.65	0.65

Abbreviations: BIA = bioelectrical impedance analysis; MSRA = Mini Sarcopenia Risk Assessment questionnaire; ROC = receiver operating characteristic; SARC-CalF = Strength, Assistance with walking, Rising from a chair, Climbing stairs, Calf circumference, and Falls questionnaire; SARC-F = Strength, Assistance with walking, Rising from a chair, Climbing stairs, and Falls questionnaire.

## Data Availability

The raw data supporting the conclusions of this article will be made available by the authors upon request.

## Abbreviations

The following abbreviations are used in this manuscript:

95% CI	95% confidence interval
ACR	American College of Rheumatology
anti-CCP	Anti-cyclic citrullinated peptide
AWGS	Asian Working Group for Sarcopenia
bDMARDs	Biological disease-modifying antirheumatic drugs
BIA	Bioelectrical impedance analysis
CC	Calf circumference
CT	Computed tomography
DAS28	Disease Activity Score-28 joint counts
DXA	Dual X-ray absorptiometry
ESR	Erythrocyte sedimentation rate
EULAR	European League Against Rheumatism
EWGSOP-2	European Working Group on Sarcopenia in Older People version 2
IQR	Interquartile range
MRI	Magnetic resonance imaging
MSRA	Mini Sarcopenia Risk Assessment questionnaires
NSAIDs	Non-steroidal anti-inflammatory drugs
RA	Rheumatoid arthritis
RF	Rheumatoid factor
ROC	Receiver operating characteristic
SARC-CalF	Strength, Assistance with walking, Rising from a chair, Climbing stairs, Calf circumference, and Falls questionnaire
SARC-F	Strength, Assistance with walking, Rising from a chair, Climbing stairs, and Falls questionnaire
